# Supplementary material for: A Tumor-Specific Molecular Network Promotes Tumor Growth in Drosophila by Enforcing a Jun N-Terminal Kinase–Yorkie Feedforward Loop
Source: Cancers (Basel). 2024 May 2;16(9):1768. doi: 10.3390/cancers16091768 (PMC11083887; doi:10.3390/cancers16091768)
Supplement: Supplementary file 1 [file cancers-16-01768-s001.zip › cancers-2928821-supplementary.pdf]

Supplementary Materials: The following supporting information can be downloaded at:

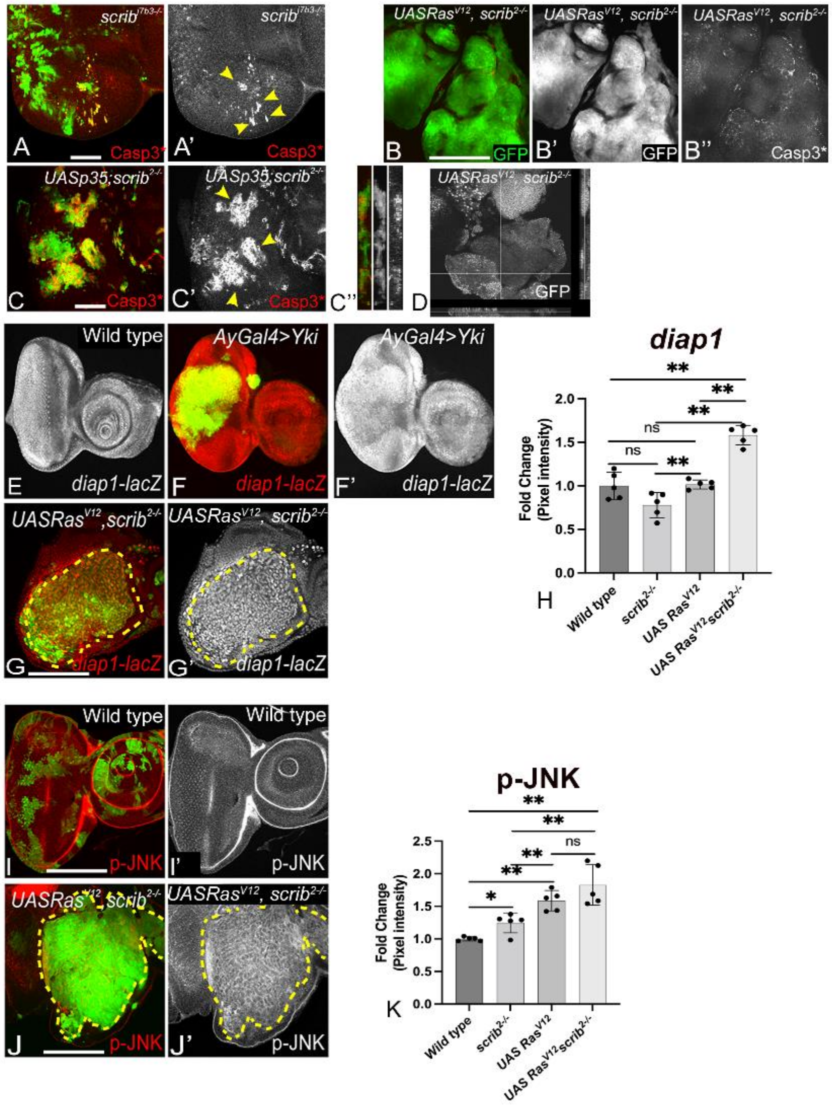

Figure S1 Related to Figure 1 Waghmare et al

Fig. S1 (related to Fig. 1): Growth of *scrib* mutant clones and the role of Yki and JNK signaling in promoting *Ras^{V12} scrib$^-$* clones. (A-D) Panels show MARCM clones in eye discs from (A, GFP, A′ grey) *scrib$^-$*, (B GFP, B′ grey) *Ras^{V12} scrib$^-$* (20X magnification of disc shown in Fig. 1 C) and (C, GFP C′ grey) *scrib$^-$,P35* stained for activated Casp 3* (A,B, C red, A′,B′,C′ grey). C′′ shows YZ sections of clones show the monolayer organization. (D) Wing disc showing *Ubx-Flp; Ras^{V12} scrib$^-$* MARCM clones that show aggressive, invasive growth. (E-H) Show expressing of *diap1-lacZ* (using anti-β-gal antibodies) in wild-type (E), flp-out clones of Yki (F red, F′ grey) and in MARCM clones of *Ras^{V12} scrib$^-$* (G red, G′ grey). The bar scatter plot in H depicts the normalized pixel intensity from indicated genotypes for expression of *diap1-lacZ*. (I-K) MARCM clones (GFP, green) of wild-type (I) and *Ras^{V12} scrib$^-$* (J) genotypes stained for pJNK (I, J red, I′, J′ grey). The bar scatter plot in K show changes in pJNK levels for the tested genotypes. (H, K) Statistical significance was calculated using Mann-Whitney test, where ns denotes *p*-value $\geq$ 0.05, and asterisks show significant interactions * *p*-value= $\leq$0.05, ** *p*-value= $\leq$0.01, *** *p*-value= $\leq$0.001 *p*-value, and **** *p*-value= $\leq$0.0001. The dashed yellow line in panels E, E′, H, H′ marks the clone boundary. Disc magnification and orientation is identical in all panels. Scale bar= 25µm

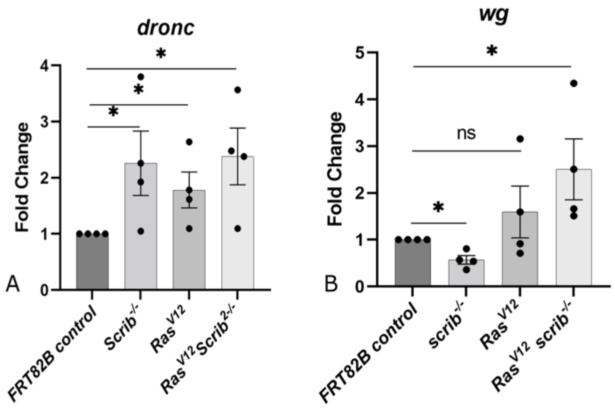

Figure S2 related to Figure 1 Waghmare et al

Fig. S2 (related to Fig. 1): Upregulation of *wg* and *dronc* expression in *Ras^{V12} scrib$^-$* clones. (A, B) graphs show fold- change in expression of *wg* (A) and *dronc* (B) in the indicated genotypes assessed by qRT-PCR (n=4). Statistical significance was calculated using Mann-Whitney test, where ns denotes *p*-value $\geq$ 0.05, and asterisks show significant interactions * *p*-value= $\leq$0.05, ** *p*-value= $\leq$0.01, *** *p*-value= $\leq$0.001 *p*-value, and **** *p*-value= $\leq$0.0001.

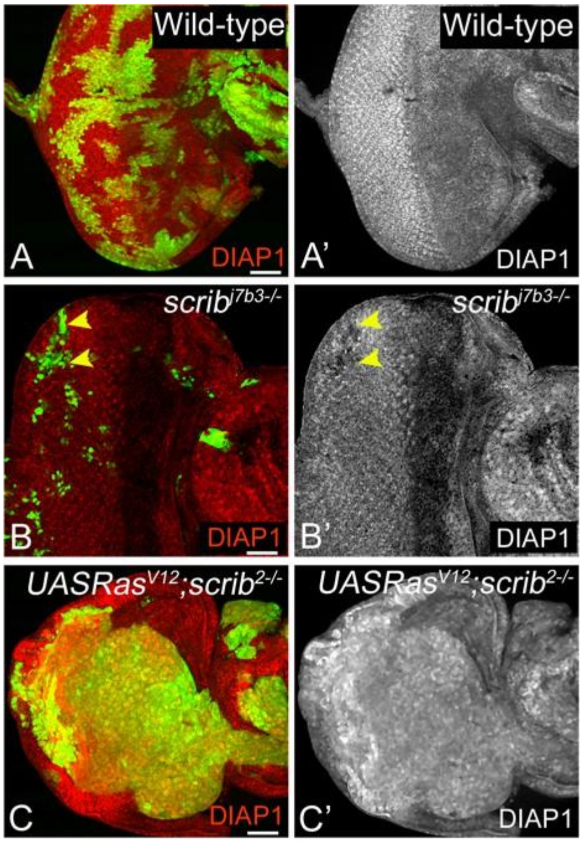

Figure S3 related to Figure2 Waghmare et al

Fig. S3 (related to Fig. 2): Assessment of cell survival in *Ras^{V12} scrib^{-}* clones. Panels show a comparison of expression of DIAP1 (red, grey) in wild-type, *scrib^{-}* and *Ras^{V12} scrib^{-}* MARCM clones (green). Yellow arrowheads indicate downregulation of DIAP1 levels in *scrib^{-}* clones (middle row). Disc magnification and orientation is identical in all panels. Scale bar= 40µm

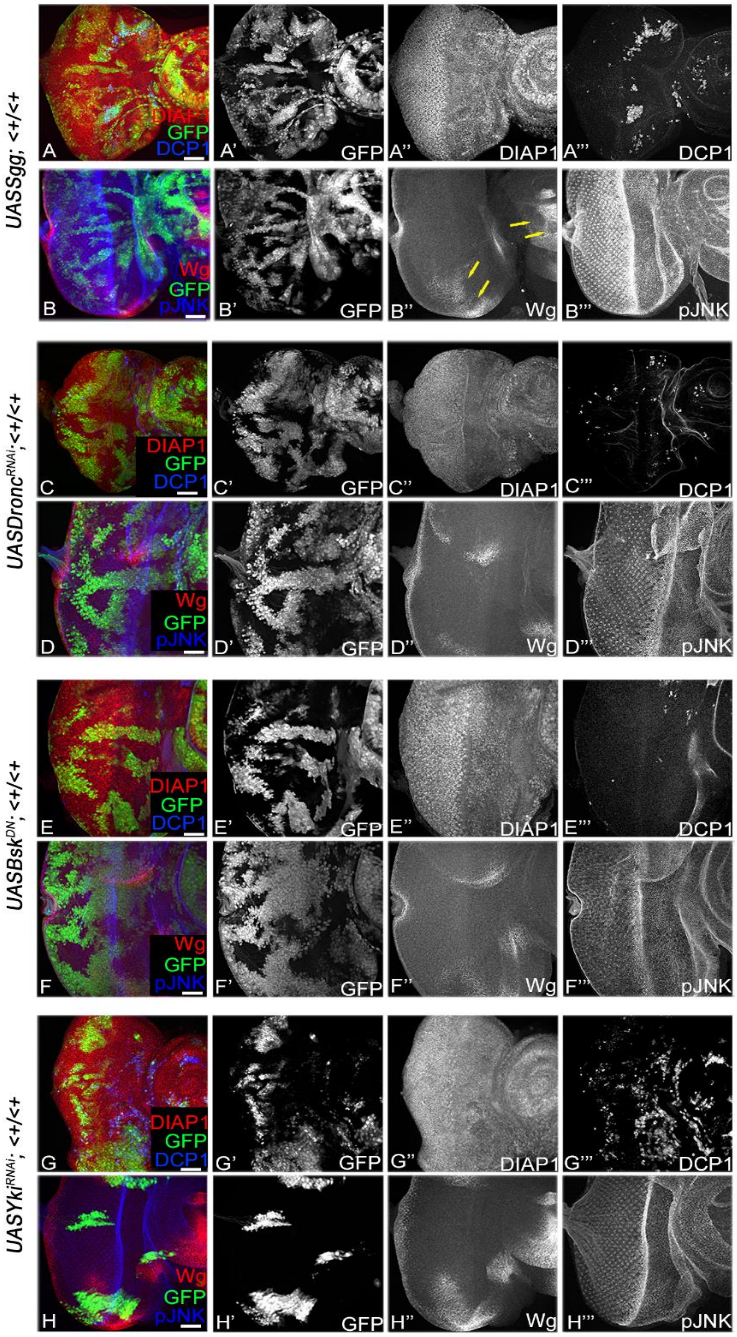

Figure S4 related to Figure 2 Waghmare et al

Fig. S4 (related to Fig. 2): Effects of Downregulation of network components in wild-type cells. Panels show MARCM clones of wild-type cells in which we depleted (A-B′′′) Wg (*UASgg*^{S9a};<+/<+), (C-D′′′) Dronc (*UASDronc*^{RNAi}; <+/<+), (E-F′′′) JNK (*UASBsk*^{DN}; <+/<+) or (G-H′′′) Yki (*UASYki*$^{N+CRNAi}$; <+/<+) and tested effects on expression of DIAP1 (A, C, E, G red, A″, C″, E″, G″ grey), DCP1 (A, C, E, G blue, A″′, C″′, E″′, G″′ grey), Wg (B, D, F, H red, B″, D″, F″, H″ grey), and pJNK (B, D, F, H blue, B″′, D″′, F″′, H″′ grey). Disc magnification and orientation is identical in all panels. Scale bar= 25μm

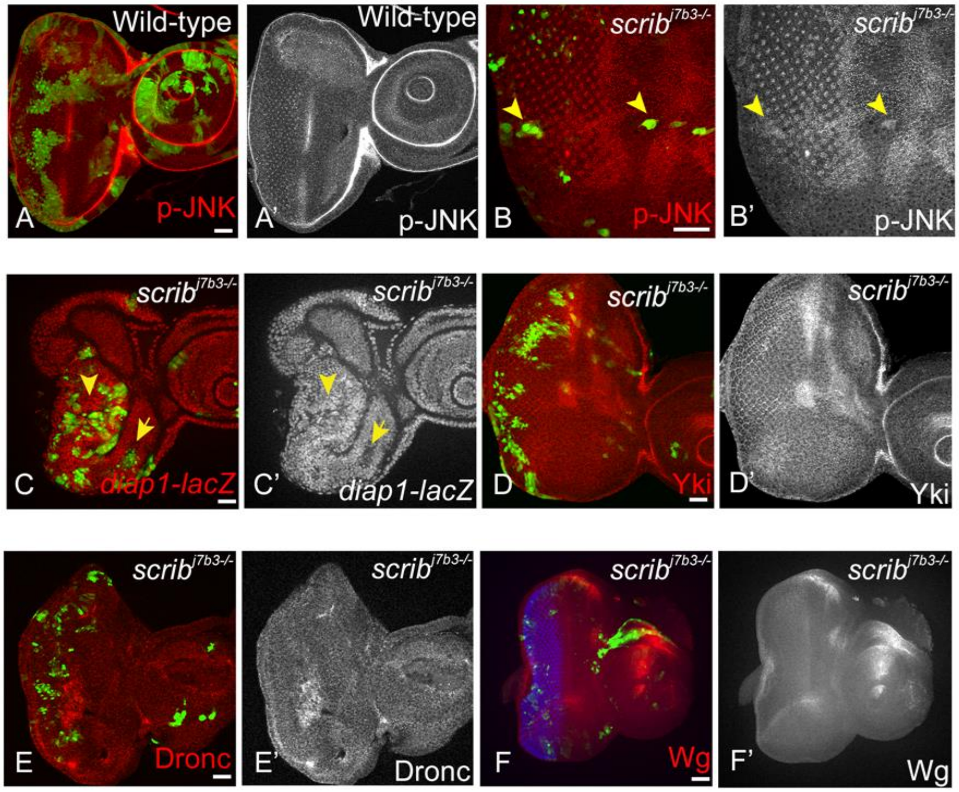

Figure S5 related to Figure 5 Waghmare et al

Fig. S5 (related to Fig. 5): Effects of loss of *scrib* on the molecular network. Panels show *scrib*⁻ MARCM clones (green) in eye imaginal discs stained to assess expression of pJNK (A, B red, A' B' grey), *diap1-lacZ* using anti-β-gal antibodies (C red, C' grey), Yki (D red, D' grey), Dronc (E red, E' grey) and Wg (F red, F' grey). Disc magnification and orientation is identical in all panels. Scale bar= 25μm

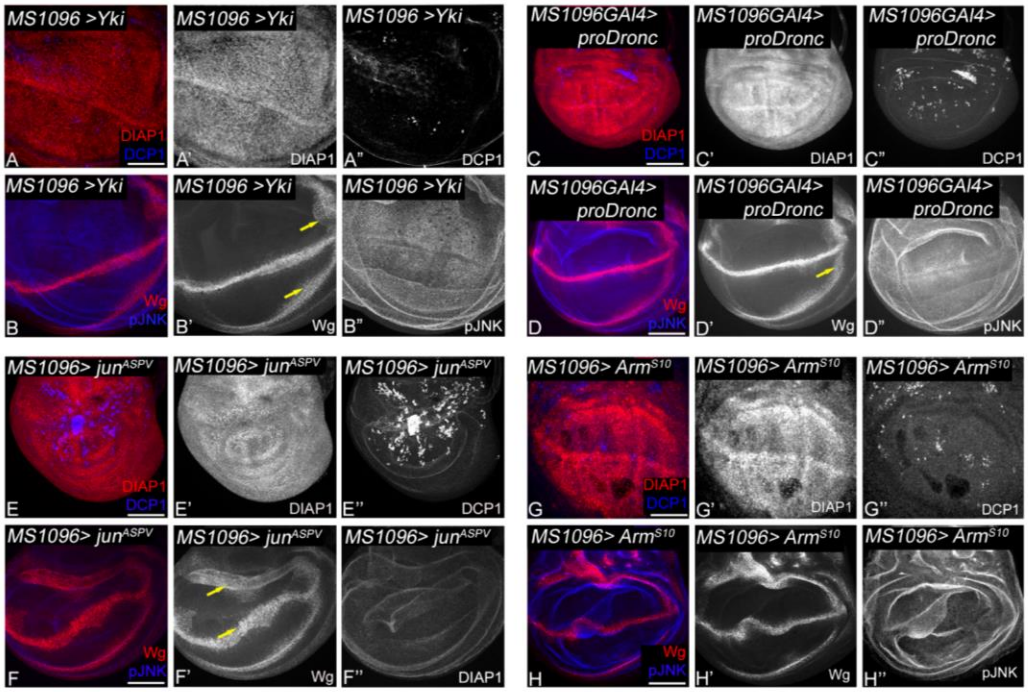

Figure S6 related to Figure 7 Waghmare et al

Fig. S6 (related to Fig. 7): Activation of individual network components in polarity intact cells cause mild effects. Panels show wing imaginal discs from (A-B") *MS1096>UAS Yki*, (C- D") *MS1096>UASproDronc*, (E-F") *MS1096>UASjun^aspv*, and (G-H") *MS1096>UASArm^S10* stained for DIAP1 (A, C, E, G red, A', C', E', G' grey), DCP1 (A, C, E, G blue, A", C", E", G" grey), Wg (B, D, F, H red, B', D', F', H' grey) and pJNK (B, D, F, H blue, B", D", F", H" grey). The yellow arrows mark induction of Wg. Disc magnification and orientation is identical in all panels. Scale bar= 25μm
